# The occurrence and extent of anxiety and distress among Dutch travellers after encountering an animal associated injury

**DOI:** 10.1186/s40794-023-00193-x

**Published:** 2023-08-15

**Authors:** Anouk M. T. Warmerdam, Floriana S. Luppino, Leo G. Visser

**Affiliations:** 1 Leiden University Medical Centre, Leiden, the Netherlands; 2 Eurocross Assistance, Leiden, the Netherlands; 3 Department of Infectious Diseases, Leiden University Medical Centre, Leiden, the Netherlands

**Keywords:** Animal associated injury, Animal bites, Rabies, Anxiety, HADS, Distress, Medical assistance organisation, Pre-exposure prophylaxis, Post-exposure prophylaxis, Travellers

## Abstract

**Background:**

Prompt administration of post-exposure prophylaxis (PEP) is crucial to prevent a fatal rabies infection after an animal associated injury (AAI), preferably within 24 h. PEP, especially in case of a type III injury for which rabies immune globulin (RIG) is needed, is difficult to obtain abroad. This, along with the fear of potentially having contracted a lethal disease, might be an important source for anxiety and distress. We investigated the occurrence and extent of self-reported anxiety and distress at different timepoints among Dutch travellers after encountering an AAI, and the involved factors.

**Methods:**

A retrospective quantitative observational study was conducted including insured Dutch travellers who actively contacted Eurocross Assistance after encountering an AAI abroad. An online questionnaire was designed to measure anxiety and distress levels, using the HADS (Hospital Anxiety and Depression Scale) and distress thermometer at three time points: departure from home (T1), post-AAI (T2), and treatment administration (T3). Statistical analyses included T-tests, Chi-square tests, and ANCOVA analyses.

**Results:**

We showed a significant increase in mean anxiety and distress scores at T2, and a significant decrease at T3. Women were more often anxious and distressed. Between T1 and T2, PrEP, and being aware of the risks were positively associated with anxiety levels, and PrEP and WHO region Africa with distress levels. Between T2 and T3, anxiety levels remained higher for monkey-induced injury, thoracic injuries, and WHO region Southeast Asia. PEP-delay between 24–48 h resulted in decreased distress levels at this time period, while type II injury elevated distress levels.

**Conclusions:**

This study showed significant anxiety and distress levels after an AAI among the vast majority of travellers, which is detrimental to their health-related quality of life (HR-QOL). This highlights the importance of proper pre-travel information. In the context of rabies prevention, these results suggest that pre-travel advice and policy makers should also take aspects of HR-QOL into consideration.

**Supplementary Information:**

The online version contains supplementary material available at 10.1186/s40794-023-00193-x.

## Background

Rabies is a fatal zoonotic viral disease once clinical symptoms show [1, 2]. Each year, rabies (RABV) causes approximately 59,000 human deaths, 95% of which occur in Asia and Africa, and 80% in rural regions. The associated global burden of disease is estimated to be 3.7 million disability-adjusted life-years (DALYs) and is predominantly caused by premature death [2–4]. RABV enters the body through wound tissue or mucosal surfaces after contact with infected saliva of a rabid animal, in 99% of the cases a dog [1, 2, 4]. The incubation period ranges from 5 days to various years, but generally first symptoms occur within 2–3 months [2].

As long as clinical symptoms are absent, there is a window of opportunity. Therefore, in case of an animal-associated injury (AAI) with apparent damaged skin barrier, the administration of post-exposure rabies prophylaxis (PEP) is crucial [1, 2]. PEP consists of thorough wound cleansing, rabies vaccine and, depending on the type of injury, also rabies immune globulin (RIG). If indicated, RIG should be administered within 24 h [1, 2]. Especially for travellers, the administration of pre-exposure prophylaxis (PrEP) is a more adequate preventive strategy. PrEP with two doses rabies vaccines provides lifelong immunological memory. In case of an AAI, only two rabies revaccinations, but no RIG, are required as PEP. See also table in the Additional file 3 [2, 5].

Although the prevalence of rabies among travellers is low, with only 63 reported infections between 1990 and 2013, travellers are often exposed to a potential infection risk when encountering an AAI abroad [6]. Current rabies prevention measures, apart from human vaccines, include dog vaccination programmes, proper wound management, and avoiding animal contact. Pre-travel advice primarily consists of the latter, along with getting PrEP when travelling to endemic countries [2, 5]. However, the percentage of unvaccinated travellers with an AAI remains over 90% [7–10]. The advice to travellers to take PrEP is based on five determinants: the endemicity of canine rabies, local access to proper medical care and PEP, duration of stay, presumed engagement in high-risk activities, and age [2, 9]. These determinants greatly differ among countries. In addition, rabies vaccines are not always available. RIG is expensive and even more scarcely available, especially in remote rural areas [4, 11, 12]. Travellers encountering an AAI abroad can therefore not be ensured of the availability of adequate PEP, especially if RIG is required. In order to acquire PEP in time, the affected individual is often required to travel to a different city or even country [13, 14]. Multiple studies found that of all AAI cases in need of RIG, only 10% received it in the original destination country [8, 10, 13]. This, along with the fear of potentially having contracted an almost universally lethal disease, can be an important source for anxiety and distress.

The fear of contracting an infectious disease has been investigated before, for instance for Lyme disease and SARS-CoV-2 [15–17]. Regarding rabies, previous research mainly focussed on exposure risk and risk factors for PEP-delay among (Dutch) travellers [10, 14]. Intuitively, a higher PrEP uptake seems a reasonable strategy to reduce risk of infection and consequent anxiety and distress. A recent cost evaluation of different rabies vaccination strategies in the Netherlands by Suijkerbuijk et al. [18] concluded, in contrast, that a higher PrEP uptake would result in higher overall costs. However, only material costs were analysed, while loss of quality of life was not taken into account. Psychological status and well-being are important domains for measuring quality of life [19, 20]. To our knowledge, this concept, in relation to rabies exposure, has never been systematically studied. This study aimed to investigate the occurrence and extent of self-reported anxiety and distress levels at different timepoints among Dutch travellers after encountering an AAI, and which factors may influence these levels. The outcomes may give insight into which factors could be addressed to improve the quality of pre-travel information in order to prevent and avoid (unnecessary) anxiety and distress.

## Methods

### Study design and population

A retrospective quantitative observational study was conducted. The study sample consisted of solely Dutch insured travellers who actively contacted Eurocross Assistance (ECA) after encountering an AAI while being abroad. Eurocross Assistance (ECA), Leiden, the Netherlands, is one of the leading medical assistance organisations in the Netherlands, assisting insured Dutch citizens abroad in need of medical support [21]. Additional ‘rabies questions’ (*i.e.* having received PrEP, type of wound) were asked to provide proper medical advice in accordance to WHO guidelines and to help locate medical treatment.

### Data collection

The original database of ECA consisted of 690 AAI notifications, providing information about the AAI (provocation, animal, injury type), PrEP status, and (local) PEP advice (rabies vaccine and/or RIG) recorded between December 2015 and February 2019. An electronic questionnaire was sent to 631 cases (91%) of whom an e-mail address was available for additional data. All participants gave their consent. No exclusion criteria were used since an AAI can happen to anyone from young to old.

The questionnaire consisted of three ‘domains’: ‘anxiety’, ‘distress’, and ‘awareness and reassurance’. These domains were evaluated at three different timepoints: time of departure from home to vacation destination (T1); immediately after AAI (T2), and the moment of PEP administration (T3). The rationale for the chosen timeframe is as follows: T1 represented baseline, the AAI and its unexpectedness shortly before T2 was thought to trigger anxiety the most, and treatment at T3 was thought to temper these levels.

The Hospital Anxiety and Depression Scale (HADS), a validated and extensively used questionnaire [22, 23], was used to assess anxiety. Since depression is beyond the scope, the HADS-A variant was selected, which contains seven self-reported items using a 4-point Likert scale ranging from 0 to 3 with a total score between 0 and 21 [24]. Different subgroups can be identified based on severity according to the HADS manual: no anxiety (≤ 7); mild (8–10), moderate (11–15), and severe anxiety(16 ≥) [23, 25].

The Distress Thermometer [17], a thermometer-like single self-reported analogue scale from 0 to 10, was used to measure distress [26]. As previously found as optimal cut-off in relation to the HADS, a score of 5 or higher indicated distress [27]. Other scales were excluded due to poor fit to the study aim or the considerable number of questions, which likely decreases the response rate. Questions about risk awareness, information about rabies aetiology, and local PEP advice were asked separately in the last domain (Additional file 1).

### Variables

Our main outcome variables were anxiety and distress scores, which are supposedly correlated with perceived risk of infection, measured at three timepoints. First, covariates were clustered based on the assumption they increased contagion risk [10, 13, 14]: animal involved, WHO region, type of injury according to WHO, location of injury, travel duration in days, travelled multiple countries, PEP-delay (within 24 h, delay between 24 and 48 h, and delay of 48 h or more), and provocation defined as an AAI by self-approach to the animal. Covariates with potential protective effects on anxiety and distress were completed PrEP (yes/no), ‘informed about the disease and its consequences’, ‘awareness of the risk of a possible bite or scratch from an animal at my destination abroad’, and medical help conform to advise from the Netherlands [7, 9, 10, 13, 14]. Age and gender were included as basic variables.

### Statistical analysis

Statistical analyses were performed using IBM SPSS Statistics version 26. Descriptive statistics were computed using means and standard deviations (SD) or percentages, as appropriate. To investigate which factors significantly differed for anxiety and distress, at both T2 and T3, Chi-square tests and T-tests were performed as appropriate [28]. To investigate which factors contributed to the increase or decrease in anxiety and distress levels between timepoints while taking the levels at the previous timepoint into account, ANCOVA were conducted.

## Results

The online questionnaire was returned by 222 travellers (35.2%), of which 167 (26.5%) were complete. Responders more often encountered PEP-delay of > 48 h (22.7%), compared to non-responders (15.4%) (*p* = 0.058) (results not shown). Participants’ general characteristics are shown in Table 1. At T2, 60.5% of travellers experienced anxiety, of which 19.2% severe levels (Additional file 2).

**Table 1:** Descriptive characteristics of the study sample (*N* = 222)

**General characteristics and anxiety and distress scores**		**N (variable)**
Age in years (mean, SD)	30.0 (13.2)	220
Number of men, N (%)	99 (44.6%)	222
Received PrEP, N (%)	66 (30.3%)	218
WHO region		222
South-East Asia, N (%)	104 (46.8%)	
Western Pacific, N (%)	37 (16.7%)	
Europe, N (%)	28 (12.6%)	
South America, N (%)	18 (8.1%)	
Africa, N (%)	13 (5.9%)	
Eastern Mediterranean, N (%)	10 (4.5%)	
Central America, N (%)	7 (3.2%)	
North America, N (%)	5 (2.3%)	
Travel duration in days (median, IQR)	25 (47.8)	200
Type of animal		221
Dog, N (%)	117 (52.9%)	
Monkey, N (%)	54 (24.4%)	
Cat, N (%)	42 (19.0%)	
Bat, N (%)	5 (2.3%)	
Rodent, N (%)	0 (0.0%)	
Other, N (%)	3 (1.4%)	
Provocation		200
Provoked AAI, N (%)	25 (12.5%)	
Unexpected AAI, N (%)	175 (87.5%)	
Location injury		220
Arm/hand area, N (%)	93 (42.3%)	
Calf/foot area, N (%)	88 (40.0%)	
Thigh, N (%)	22 (10.0%)	
Head/neck area, N (%)	11 (5.0%)	
Thorax, N (%)	4 (1.8%)	
Other, N (%)	2 (0.9%)	
WHO classification wound		215
Type I, N (%)	15 (7.0%)	
Type II, N (%)	58 (27.0%)	
Type III, N (%)	142 (66.0%)	
HADS score^1^		
T1, mean (SD)	2.4 (2.7)	177
T2, mean (SD)	9.6 (5.7)	177
T3, mean (SD)	6.6 (5.4)	145
DT score^2^		
T1, mean (SD)	1.3 (2.0)	167
T2, mean (SD)	6.4 (2.7)	167
T3, mean (SD)	4.6 (3.1)	140
Informed about rabies at T1, N (%)	100 (57.1%)	175
Informed about rabies at T2, N (%)	130 (74.3%)	175
Delay PEP		181
No delay, N (%)	95 (52.5%)	
Delay under 48 h, N (%)	45 (24.9%)	
Delay over 48 h, N (%)	41 (22.7%)	
Received treatment according to advice, N (%)	125 (71.8%)	174

List of abbreviations: *N* Number of valid cases in total, *SD* Standard deviation, *IQR* Inter-quartile range, *HADS* Hospital Anxiety and Depression Scale, *DT* Distress Thermometer, *AAI* Animal Associated Injury, *PrEP* Pre-Exposure Prophylaxis, *WHO* World Health Organisation, *PEP* Post-Exposure Prophylaxis

^1, 2^The number of valid cases for the scores of the HADS at T3 was *N* = 145 and for the DT at T3 *N* = 140, thereby deviating from the numbers at T1 and T2 as for some travellers no further medical action was required. Higher HADS and DT scores indicate higher anxiety and distress levels. The maximum score for HADS is 21, for DT the maximum score is 10

**Fig. 1:**
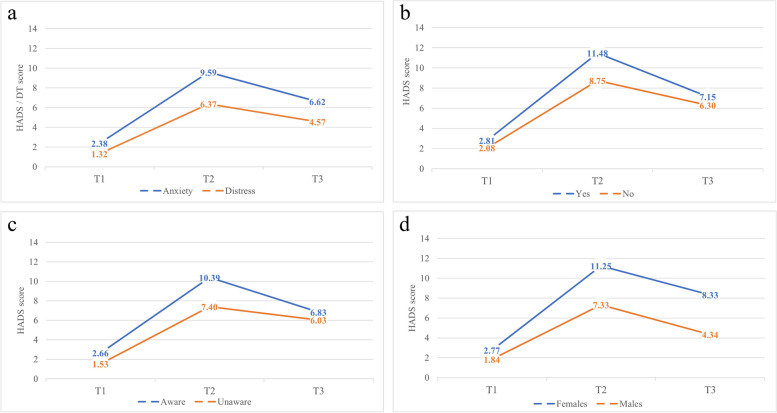
Mean anxiety and distress scores, and anxiety scores according to having received PrEP, presence of risk awareness, and gender between timepoints^1^. **a** Mean anxiety vs distress. **b** Mean anxiety for PrEP. **c** Mean anxiety for risk awareness. **d** Mean anxiety for gender. ^1^The maximum score for HADS is 21, for DT the maximum score is 10. Higher scores indicate higher levels of anxiety/distress

Figure 1a shows the significant increase in mean anxiety and distress scores at T2, and the significant decrease at T3 (*p* =  < 0.001). Mean anxiety and distress levels also differed significantly between T1 and T3 (both *p* =  < 0.001). Figure 1b-d show mean anxiety scores between timepoints for PrEP, risk awareness, and gender respectively.

At T2, females were more often anxious and distressed than males; travellers with PrEP were more often distressed, and those aware of the risks were more often anxious (Table 2). At T3, females were again more anxious and distressed and those informed at T2 were less anxious.

**Table 2:** Variables according to the presence of anxiety and distress at timepoint 2 and 3

	**Anxiety, timepoint 2**			**Anxiety, timepoint 3**			**Distress, timepoint 2**			**Distress, timepoint 3**		
	Anxiety	No anxiety	*P*- value	Anxiety	No anxiety	*P*-value	Distress	No distress	*P*-value	Distress	No distress	*P*-value
Age (mean)	105 (29.4)	70 (32.1)	.21	49 (29.1)	95 (28.5)	.74	124 (30.0)	41 (32.0)	.47	74 (29.0)	66 (28.6)	.85
Gender			** < .001**			** < .001**			**.008**			**.005**
Male	31 (41.3%)	44 (58.7%)		9 (14.5%)	53 (85.5%)		47 (65.3%)	25 (34.7%)		24 (39.3%)	37 (60.7%)	
Female	76 (74.5%)	26 (25.5%)		40 (48.8%)	42 (51.2%)		79 (83.2%)	16 (16.8%)		50 (63.3%)	29 (36.7%)	
Received PrEP			.14			.09			**.009**			.86
Yes	37 (68.5%)	17 (31.5%)		20 (43.5%)	26 (56.5%)		45 (88.2%)	6 (11.8%)		23 (51.1%)	22 (48.9%)	
No	68 (56.7%)	52 (43.3%)		28 (29.2%)	68 (70.8%)		78 (69.0%)	35 (31.0%)		49 (52.7%)	44 (47.3%)	
Risk awareness T1			**.024**			.072			.068			.97
Yes	86 (65.6%)	45 (34.4%)		42 (38.2%)	68 (61.8%)		98 (79.0%)	26 (21.0%)		57 (52.8%)	51 (47.2%)	
No	21 (46.7%)	24 (53.5%)		7 (21.2%)	26 (78.8%)		28 (65.1%)	15 (34.9%)		17 (53.1%)	15 (46.9%)	
Informed at T1			.66			.33			.51			.31
Yes	62 (62.0%)	38 (38.0%)		27 (31.4%)	59 (68.6%)		75 (77.3%)	22 (22.7%)		42 (49.4%)	43 (50.6%)	
No	44 (58.7%)	31 (41.3%)		22 (39.3%)	34 (60.7%)		51 (72.9%)	19 (27.1%)		32 (58.2%)	23 (41.8%)	
Informed at T2						**.019**						.51
Yes				32 (29.4%)	77 (70.6%)					56 (51.4%)	53 (48.6%)	
No				17 (51.5%)	16 (48.5%)					18 (58.1%)	13 (41.9%)	
Provocation			0.68			.46			.57			.083
Provoked	15 (78.9%)	4 (21.1%)		4 (26.7%)	11 (73.3%)		14 (82.4%)	3 (17.6%)		4 (30.8%)	9 (69.2%)	
Unexpected	81 (57.0%)	61 (43.0%)		43 (36.4%)	75 (63.6%)		102 (74.5%)	35 (25.5%)		65 (56.0%)	51 (44.0%)	
Type III injury			.67			.95			.85			.61
Yes	68 (59.1%)	47 (40.9%)		34 (34.7%)	64 (65.3%)		85 (75.9%)	27 (24.1%)		52 (53.6%)	45 (46.4%)	
No	35 (62.5%)	21 (37.5%)		14 (34.1%)	27 (65.9%)		38 (74.5%)	13 (25.5%)		19 (48.7%)	20 (51.3%)	
Reassured at T2						.45						.76
Yes				31 (32.6%)	64 (67.4%)					50 (53.8%)	43 (46.2%)	
No				18 (39.1%)	28 (60.9%)					24 (51.1%)	23 (48.9%)	
Location injury			.89			.52			.65			.90
Head/neck area	3 (75.0%)	4 (57.1%)		0 (0.0%)	4 (100.0%)		4 (57.1%)	3 (42.9%)		2 (50.05)	2 (50.0%)	
Thorax	45 (61.5%)	1 (25.0%)		2 (66.7%)	1 (33.3%)		4 (100.0%)	0 (0.0%)		2 (66.7%)	1 (33.3%)	
Arm/hand area	12 (66.7%)	28 (38.4%)		19 (32.8%)	39 (67.2%)		50 (76.9%)	15 (23.1%)		25 (46.3%)	29 (53.7%)	
Thigh	43 (58.9%)	6 (33.3%)		4 (26.7%)	11 (73.3%)		12 (75.0%)	4 (25.0%)		8 (57.1%)	6 (42.9%)	
Calf-foot area	1 (50.0%)	30 (41.1%)		23 (37.1%)	39 (62.9%)		54 (74.0%)	19 (26.0%)		36 (57.1%)	27 (42.9%)	
Other	3 (42.9%)	1 (50.0%)		1 (50.0%)	1 (50.0%)		2 (100.0%)	0 (0.0%)		1 (50.0%)	1 (50.0%)	
Type of animal			.84			.44			.26			.24
Dog	58 (61.7%)	36 (38.3%)		25 (32.9%)	51 (67.1%)		72 (80.0%)	18 (20.0%)		42 (56.0%)	33 (44.0%)	
Cat	18 (56.3%)	14 (43.8%)		6 (25.0%)	18 (75.0%)		20 (66.7%)	10 (33.3%)		8 (32.0%)	17 (58.0%)	
Monkey	27 (62.8%)	16 (37.2%)		16 (42.1%)	22 (57.9%)		30 (75.0%)	10 (25.0%)		21 (60.0%)	14 (40.0%)	
Bats	2 (50.0%)	2 (50.0%)		0 (0.0%)	3 (100.0%)		2 (66.7%)	1 (33.3%)		1 (50.0%)	1 (50.0%)	
Other	1 (33.3%)	2 (66.7%)		1 (50.0%)	1 (50.0%)		1 (33.3%)	2 (66.7%)		1 (50.0%)	1 (50.0%)	

Table 3 shows results of ANCOVA analyses. Anxiety levels between T1 and T2 were positively associated with the female gender, having received PrEP, and being aware of the risks. Additionally, anxiety levels were elevated by WHO regions Africa and Central America compared to Europe. Between T2 and T3, anxiety levels generally decrease. However, T3 anxiety levels remained higher for females, monkey-induced injury compared to a dog, thoracic injuries compared to upper limb injuries, and WHO region Southeast Asia compared to Europe.

**Table 3:** Results of ANCOVA analyses for anxiety and distress levels between timepoints and each covariate added separately

	**Anxiety**						**Distress**					
	**T1 & T2**			**T2 & T3**			**T1 & T2**			**T2 & T3**		
**Variable**	**B**	**SE**	**95%-CI**	**B**	**SE**	**95%-CI**	**B**	**SE**	**95%-CI**	**B**	**SE**	**95%-CI**
Gender (male (0), female (1))	3.25	0.78	**1.71; 4.78**	1.42	0.69	**0.05; 2.79**	1.21	0.40	**0.42; 1.99**	0.86	0.49	-0.10; 1.82
Age			*P* < 0.001			*P* < 0.001			*P* = 0.001			*P* < 0.001
< 23 years	Ref			Ref			Ref			Ref		
23.1–27.5 years	0.69	1.13	-1.54; 2.91	0.87	0.88	-0.87; 2.60	0.68	0.57	-0.45; 1.80	0.41	0.64	-0.87; 1.68
27.5–34.5 years	0.95	1.11	-1.24; 3.13	-0.75	0.88	-2.50; 1.00	0.59	0.56	-0.52; 1.70	-0.27	0.64	-1.56; 1.01
34.6 > years	-0.60	1.11	-2.79; 1.58	0.39	0.95	-1.48; 2.25	-0.14	0.56	-1.25; 0.97	0.37	0.68	-0.98; 1.72
PrEP (yes (1), no(0))	2.12	0.86	**0.43; 3.81**	-0.72	0.70	-2.10; 0.66	1.16	0.43	**0.31; 2.02**	-0.48	0.51	-1.49; 0.54
Type of injury			*P* < 0.001									
Type I	Ref			Ref			Ref			Ref		
Type II	-2.39	1.71	-5.78; 0.99	-3.09	2.29	-7.62; 1.44	-0.32	0.97	-2.23; 1.59	-3.98	2.00	**-7.94; -0.02**
Type III	-2.38	1.60	-5.55; 0.78	-2.59	2.24	-7.01; 1.83	-0.37	0.91	-2.17; 1.43	-4.00	1.97	**-7.90; -0.10**
Risk awareness T1 (yes (1), no(0))	2.14	0.91	**0.34; 3.93**	-0.28	0.78	-1.82; 1.26	0.69	0.46	-0.22; 1.61	-0.21	0.56	-1.32; 0.91
Unexpected AAI (yes (1), no(0))	-0.73	1.32	-3.34; 1.88	1.79	1.05	-0.29; 3.87	0.04	0.67	-1.29; 1.36	0.90	0.82	-0.72; 2.51
Delay PEP			*P* < 0.001			*P* < 0.001			*P* < 0.001			*P* < 0.001
< 24 h	Ref			Ref			Ref			Ref		
24 h – 48 h	1.01	1.06	-1.09; 3.10	-0.78	0.79	-2.34; 0.78	0.59	0.51	-0.42; 1.59	-1.30	0.57	**-2.42; -0.18**
48 h >	-0.06	1.12	-2.27; 2.16	0.80	0.84	-0.86; 2.45	0.27	0.53	-0.79; 1.32	0.16	0.60	-1.01; 1.34
Type of animal			*P* < 0.001			*P* < 0.001			*P* = 0.002			*P* < 0.001
Dog	Ref			Ref			Ref			Ref		
Cat	-1.07	1.08	-3.21; 1.07	0.70	0.87	-1.03; 2.42	-0.55	0.55	-1.64; 0.54	-0.56	0.65	-1.85; 0.72
Monkey	-0.18	0.98	-2.10; 1.75	1.81	0.74	**0.34; 3.28**	-0.33	0.50	-1.31; 0.65	0.63	0.57	-0.49; 1.76
Bat	-0.17	2.71	-5.51; 5.17	-2.21	2.22	-6.59; 2.17	-0.35	1.54	-3.39; 2.70	-0.31	1.99	-4.24; 3.62
Other	-3.86	3.12	-10.02; 2.30	4.28	2.69	-1.04; 9.61	-1.84	1.54	-4.88; 1.20	1.36	2.02	-2.62; 5.35
Location of injury			*P* < 0.001			*P* < 0.001			*P* = 0.001			*P* < 0.001
Arm/hand area	Ref			Ref			Ref			Ref		
Head/neck area	-0.91	2.10	-5.06; 3.24	-1.89	1.97	-5.78; 2.01	1.22	1.03	-3.24; 0.81	1.02	1.46	-1.86; 3.91
Thorax	4.49	2.72	-0.87; 9.85	6.28	2.26	**1.81; 10.75**	2.35	1.33	-0.28; 1.18	2.34	1.67	-0.97; 1.93
Thigh	0.43	1.40	-2.32; 3.19	-0.99	1.11	-3.18; 1.19	-0.24	0.72	-1.67; 1.18	0.26	0.84	-1.40; 1.93
Calf/foot area	-0.20	0.88	-1.93; 1.53	0.22	0.70	-1.16; 1.59	0.14	0.44	-0.73; 1.01	0.50	0.53	-0.54; 1.54
Other	-0.06	3.79	-7.54; 7.42	3.27	2.73	-2.13; 8.66	2.79	1.86	-0.88; 6.45	-0.25	2.03	-4.26; 3.76
Informed at T1 (yes (1), no(0))	-0.04	0.81	-1.62; 1.55	1.12	0.66	-0.19; 2.43	-0.45	0.41	-1.06; 0.56	0.86	0.48	-0.08; 1.81
Informed at T2 (yes (1), no(0))				-0.24	0.80	-1.82; 1.34				-0.11	0.59	-1.27; 1.04
Travel duration in days	0.01	0.01	-0.01; 0.02	0.00	0.01	-0.01; 0.01	0.00	0.00	-0.01; 0.01	0.00	0.00	-0.01; 0.01
WHO region			*P* < 0.001			*P* < 0.001			*P* < 0.001			*P* < 0.001
EU	Ref			Ref			Ref			Ref		
WP	2.41	1.47	-0.48; 5.30	1.07	1.31	-1.53; 3.66	1.18	0.73	-0.27; 2.62	0.01	0.96	-1.89; 1.92
SEA	1.54	1.25	-0.92; 4.00	2.41	1.17	**0.11; 4.71**	0.54	0.63	-0.69; 1.78	1.08	0.85	-0.61; 2.76
SA	2.73	1.73	-0.68; 6.15	0.24	1.49	-2.70; 3.19	1.28	0.86	-0.42; 0.98	0.09	1.09	-2.06; 2.24
A	5.02	1.99	**1.09; 8.95**	2.20	1.83	-1.43; 5.83	2.96	1.01	**0.95; 4.96**	0.19	1.34	-2.45; 2.83
EM	0.38	2.14	-3.84; 4.61	2.48	1.90	-1.28; 6.25	0.96	1.06	-1.13; 3.05	1.31	1.39	-1.43; 4.06
CA	4.86	2.40	**0.13; 9.58**	2.43	2.21	-1.94; 6.79	0.11	1.27	-2.40; 2.61	1.24	1.60	-1.93; 4.41
NA	1.07	2.58	-4.01; 9.58	0.10	2.47	-4.78; 4.99	1.87	1.27	-0.64; 4.38	-0.02	1.80	-3.57; 3.54
Travelled multiple countries (yes (1), no(0))	0.21	1.32	-2.41; 2.84	0.41	0.98	-1.55; 2.36	0.57	0.61	-0.65; 1.78	0.33	0.65	-0.96; 1.63

List of abbreviations: *95%-CI* 95% Confidence Intervals, *PrEP* Pre-Exposure Prophylaxis, *PEP* Post-Exposure prophylaxis, *Ref.* Reference group, *AAI* Animal Associated Injury, *WHO* World Health Organisation, *SEA* Southeast Asia, *WP* Western Pacific, *EU* Europe, *SA* South America, *A* Africa, *EM* Eastern Mediterranean, *NA* North America, *CA* Central America

Distress levels between T1 and T2 were positively affected also by female gender, having received PrEP, and WHO region Africa compared to Europe. Similar to anxiety, distress levels generally decrease between T2 and T3 but were elevated less for those with type II injury compared to type I. Distress levels were reduced by a PEP-delay between 24–48 h compared to no delay (< 24 h).

## Discussion

In this study we investigated the levels of anxiety and distress in travellers with an AAI over time, and identified which factors influenced these levels. Travellers experienced significant amounts of anxiety and distress after the AAI, especially women. PrEP, risk awareness, and WHO regions Africa and Central America were positively associated with increased anxiety directly post-AAI. After treatment, monkey-induced injury and injury in thoracic area, and region Southeast Asia were associated with less decrease in anxiety levels. Distress levels were positively associated with PrEP and WHO region Africa post-AAI, and after treatment with having a type II injury. A delay between 24 and 48 h was associated with more decrease in distress levels after treatment.

Uncertainty caused by sudden changes in everyday life may result in fear and anxiety. It is a known risk factor for significantly affecting mental health [29, 30]. In this study, a sudden event like an AAI caused anxiety among 60.5% of travellers, of which 19.2% reported severe levels. According to DSM-5, symptoms should persist for a longer time period to diagnose an anxiety disorder [31]. Although anxiety among travellers did not last long enough to be defined as a disorder, the burden did not disappear after treatment: the levels after treatment were still significantly higher compared to baseline (T1). Anxiety can have a profound impact as it is known to affect one’s behaviour, physiological and cognitive well-being [32], which are part of various quality of life (QOL) domains [32–35].

Nowadays, healthcare continues to evolve on many domains, resulting in a broader and more holistic definition of health. Health is more than absence of disease, it encompasses a wide range of contexts that cannot be expressed in money [36], emphasising the importance of health-related QOL (HR-QOL). Suijkerbuijk et al. [18] published a cost–benefit analysis for different rabies vaccination strategies in the Netherlands, but were unable to incorporate the cost implications in terms of HR-QOL. Such cost implications would be a valuable addition to the determination of health in contemporary society. Along with the increasing pressure on worldwide healthcare systems and expensive healthcare, the importance of HR-QOL is rising, thereby highlighting the significance of this study.

In line with previous research, women more often experienced anxiety and distress post-AAI. Previous research repeatedly demonstrated women to be more prone to develop anxiety and related mood disorders compared to men [37–41]. This trend is also visible through other health indicators given that women have more negative self-assessments of health, higher rates of sick leave at work, and make greater use of health services [42–44].

Having received PrEP resulted in higher anxiety and distress levels post-AAI counter to our expectations. Risk awareness also resulted in higher anxiety levels after the AAI. Having received PrEP could be accompanied with being aware of the risks of contracting rabies due to a visit to the vaccination clinic, which suggests that these factors are correlated. However, although not significant, the slope of the decrease at T3 is steeper for travellers with PrEP and risk awareness. This might suggest that even though anxiety levels for both factors spiked directly post-AAI, they also had a more reassuring effect than those without PrEP and awareness. Future studies should further investigate why travellers with PrEP are more inclined to anxiety.

In comparison to Europe, encountering an AAI in the WHO regions Africa and Central America was associated with increased anxiety after the AAI. After treatment, travellers to Southeast Asia had increased anxiety compared to those in Europe. This is in line with the assumption that not receiving RIG is associated with increased anxiety. It is known that RIG is difficult to obtain especially in Latin America and Southeast Asia. Travellers often need to travel to another country to receive RIG on time [8, 13], which increases the fear of risking a lethal infection.

Despite the sample size, contrary to our expectations, travellers with type II instead of type III had higher distress levels between AAI and treatment in comparison to type I. Although not significant, most monkey-induced injuries were type II, and none of the monkey-induced injuries was provoked. Travellers injured by monkeys compared to dogs were also more distressed. In Europe, monkeys only live in zoos. European travellers may therefore not intrinsically be aware of their potentially harmful behaviour and infections they may spread. Additionally, anxiety may be triggered even more in case of an AAI induced by a monkey, compared to one caused by a dog, which may be experienced as more familiar even for people without pets. This possibly also applies to bats but little travellers were injured by bats so no association could be found.

Interestingly, no elevated anxiety or distress levels were found for those with a PEP-delay. We found an association between PEP-delay of one day in comparison to no delay with more decrease in distress levels after treatment. Possibly, the decrease of distress levels is a natural phenomenon and given that distress was documented for a longer time period for those with PEP-delay between AAI and treatment, lower levels are to be expected. Nevertheless, this is in contradiction with other studies’ results who found increased distress in relation to treatment delay [45, 46]. A possible explanation could be a difference in risk awareness, but such analyses were off scope.

Our results imply that current pre-travel information and advice is no longer appropriate for the target group. Avoiding animal contact is fundamental to prevent rabies. However, nearly 90% of the travellers in this study encountered an AAI without provocation. Travellers with unexpected AAIs seem to be more anxious and distressed at T3, although not significant. As mentioned before, anxiety and fear are a result of uncertainty by a sudden event [30]. Stress often causes cognitive narrowing, hindering the patient from taking proper actions [47]. This could explain the spikes in anxiety and distress levels at T2 for travellers with PrEP, and also highlights the importance of pre-travel information. We believe that properly informing travellers about an AAI, its impact, the often limited availability of vaccines, and the required actions will give them a sense of control over the situation. Besides, psychological distress is known to be a wide concept which covers many emotions and psychiatric symptoms like depression and anxiety [48], suggesting that distress could be a precursor of anxiety. Tailoring information with the purpose of reducing or preferably preventing distress, and thereby anxiety, should avoid clinical levels [49]. This would be beneficial in terms of HR-QOL.

It is important that such information is received in the country of origin to reduce unnecessary distress. Apart from cultural differences, European travellers often speak different languages and are uncertain about local health care standards [47]. Information provided abroad might therefore not be fully understood, and local advice often differed from WHO guidelines [14]. Yet, the PrEP uptake in the Netherlands, indirectly referring to being informed, is relatively low [7–10]. Wieten et al. [10] found the costs and the limited time between consultation and departure to be prominent barriers in the decision-making process of PrEP. Expanding the window of time between consultation and departure might positively contribute to this decision-making process.

As for the costs, as pointed out earlier, health is no longer limited to absence of disease, but to a vaster concept of also mental health and lack of distress. Increasing awareness by improving pre-travel information might not give direct economic benefit, but is likely to increase HR-QOL. Since especially women suffer from anxiety and distress, prevention of those levels, or at least decrease, might also positively contribute to economisation by reducing sick leave and visits to healthcare providers. The experienced amount of anxiety and distress is barely expressible in an economic measure. Its lack, however, seems priceless.

### Strengths and limitations

This study is unique in addressing distress and anxiety in terms of a possible rabies infection due to an AAI. Self-reported anxiety and distress levels were measured by widely used and accepted measurement scales. Although we measured an anxious state of mind rather than a disorder, we were able to give insight into the impact of an AAI and how that relates to QOL. We believe that a simple intervention such as more targeted pre-travel information will prevent clinical anxiety levels.

Our study was limited in the sample size due to a relatively low and inconsistent response. This might be due to the unfortunate timing that coincided with the onset of the SARS-CoV-2 outbreak in the Netherlands. The AAI occurred up to four years ago at the time the questionnaire was dispatched. For this reason, recall bias might have overestimated our results as people often remember negative events more vividly [50]. Studying anxiety levels a few weeks after treatment, or a prospective study with multiple nationalities could have provided more accurate measures of the increase and decrease in anxiety. Last, although it does not yet exist, a measurement scale that measures a short-term state of mind would have matched the study better.

### Future studies

We recommend replicating this study prospectively with a different scale to measure state of mind with a larger study population, and a longer follow-up period after treatment administration. We think this will increase the response rate, and gives the opportunity to investigate more associations. It would be insightful to be able to measure QOL at baseline and after the AAI as well. This would provide opportunities for calculating intangible costs related to an AAI. Furthermore, we highlight the importance of targeted information before travelling, which effects could be studied in an intervention study.

## Conclusion

The vast majority of Dutch travellers experienced anxiety after the AAI. Anxiety was higher for females, PrEP, risk awareness, Africa, Central America, Southeast Asia, injury by monkey and in thorax area. Distress increased for females, PrEP, Africa and type II injury, and reduced by a delay between 24 and 48 h. This study’s results offer leads for tailor-made pre-travel information and advice, also for policy makers, which in turn improves travellers’ HR-QOL.

### Supplementary Information

**Additional file 1.** Questionnaire used for this study.

**Additional file 2.** Categorical HADS scores at different time points.

**Additional file 3.** Treatment scheme post-AAI.

## Data Availability

The dataset used and analysed during the current study is available upon reasonable request.

## Abbreviations

AAI: Animal Associated Injury
ANCOVA: Analysis of Covariance
DALYs: Disability Adjusted Life Years
DT: Distress Thermometer
ECA: Eurocross Assistance
HADS: Hospital Anxiety and Depression Scale
HR-QOL: Health-related quality of life
LUMC: Leiden University Medical Centre
PEP: Post-Exposure Prophylaxis
PrEP: Pre-Exposure Prophylaxis
QOL: Quality of life
RABV: Rabies virus
RIG: Rabies Immune Globulin
SARS-CoV-2: Severe acute respiratory syndrome coronavirus 2
VU: Vrije Universiteit Amsterdam (Free University Amsterdam)
WHO: World Health Organisation
