# Supplementary material for: The occurrence and extent of anxiety and distress among Dutch travellers after encountering an animal associated injury
Source: Trop Dis Travel Med Vaccines. 2023 Aug 15;9:11. doi: 10.1186/s40794-023-00193-x (PMC10426805; doi:10.1186/s40794-023-00193-x)
Supplement: Supplementary file 1 — Additional file 1. Questionnaire used for this study. [file 40794_2023_193_MOESM1_ESM.docx]

*Additional file 1. Questionnaire used for this study.*

Dear participant,

We would like to ask you to fill in the following items as accurate as possible at three different time points. Time point 1 is the moment of departure, time point 2 the moment or day directly after the animal-associated incident, and time point 3 is the moment when treatment was administered.

It is important to go back to that specific trip where you encountered the AAI and to answer the questions based on how you felt at these three specific time points.

Please note: in case treatment was deemed unnecessary from the Netherlands, you can ignore time point 3 as it does not apply.

|  | | **Time point 1:**  **Moment of departure** | **Time point 2:**  **Directly after the incident** | **Time point 3:**  **Start of treatment** |
| --- | --- | --- | --- | --- |
| 1 | I feel tense or ‘wound up’ | O – most of the time  O – a lot of the time  O – from time to time, occasionally  O – not at all | O – most of the time  O – a lot of the time  O – from time to time, occasionally  O – not at all | O – most of the time  O – a lot of the time  O – from time to time, occasionally  O – not at all |
| 2 | I get a sort of frightened feeling as if something awful is about to happen | O – very definitely and quite badly  O – yes, but not too badly  O – a little, but it doesn’t worry me  O – not at all | O – very definitely and quite badly  O – yes, but not too badly  O – a little, but it doesn’t worry me  O – not at all | O – very definitely and quite badly  O – yes, but not too badly  O – a little, but it doesn’t worry me  O – not at all |
| 3 | Worrying thoughts go through my mind | O – a great deal of the time  O – a lot of the time  O – from time to time, but not too often  O – only occasionally | O – a great deal of the time  O – a lot of the time  O – from time to time, but not too often  O – only occasionally | O – a great deal of the time  O – a lot of the time  O – from time to time, but not too often  O – only occasionally |
| 4 | I can sit at ease and feel relaxed | O – definitely  O – usually  O – not often  O – not at all | O – definitely  O – usually  O – not often  O – not at all | O – definitely  O – usually  O – not often  O – not at all |
| 5 | I get a sort of frightened feeling like 'butterflies' in the stomach | O – not at all  O – occasionally  O – quite often  O – very often | O – not at all  O – occasionally  O – quite often  O – very often | O – not at all  O – occasionally  O – quite often  O – very often |
| 6 | I feel restless as I have to be on the move | O – very much indeed  O – quite a lot  O – not very much  O – not at all | O – very much indeed  O – quite a lot  O – not very much  O – not at all | O – very much indeed  O – quite a lot  O – not very much  O – not at all |
| 7 | I get sudden feelings of panic | O – very often indeed  O – quite often  O – not very often  O – not at all | O – very often indeed  O – quite often  O – not very often  O – not at all | O – very often indeed  O – quite often  O – not very often  O – not at all |

The second part of the questionnaire is about the emotional aspect of the scratch and/or bite incident.

Like before, it is important to go back in thought to the specific trip and answer these questions based on how you felt at the three given time points.

|  | | **Time point 1:**  **Moment of departure** | **Time point 2:**  **Directly after the incident** | **Time point 3:**  **Start of treatment** |
| --- | --- | --- | --- | --- |
| 8 | Did the incident impact your emotions? |  | O – Yes  O – No | O – Yes  O – No |
| 9 | Have you had trouble with remembering things? | O – Yes  O – No | O – Yes  O – No | O – Yes  O – No |
| 10 | Did you experience problems with your self-confidence? | O – Yes  O – No | O – Yes  O – No | O – Yes  O – No |
| 11 | Did you experience anxiety? | O – Yes  O – No | O – Yes  O – No | O – Yes  O – No |
| 12 | Did you experience a tense feeling? | O – Yes  O – No | O – Yes  O – No | O – Yes  O – No |
| 13 | Did you feel lonely? | O – Yes  O – No | O – Yes  O – No | O – Yes  O – No |
| 14 | Did you experience problems with concentrating? | O – Yes  O – No | O – Yes  O – No | O – Yes  O – No |
| 15 | Have you felt guilty? | O – Yes  O – No | O – Yes  O – No | O – Yes  O – No |
| 16 | Have you experienced loss of control? | O – Yes  O – No | O – Yes  O – No | O – Yes  O – No |
| 17 | 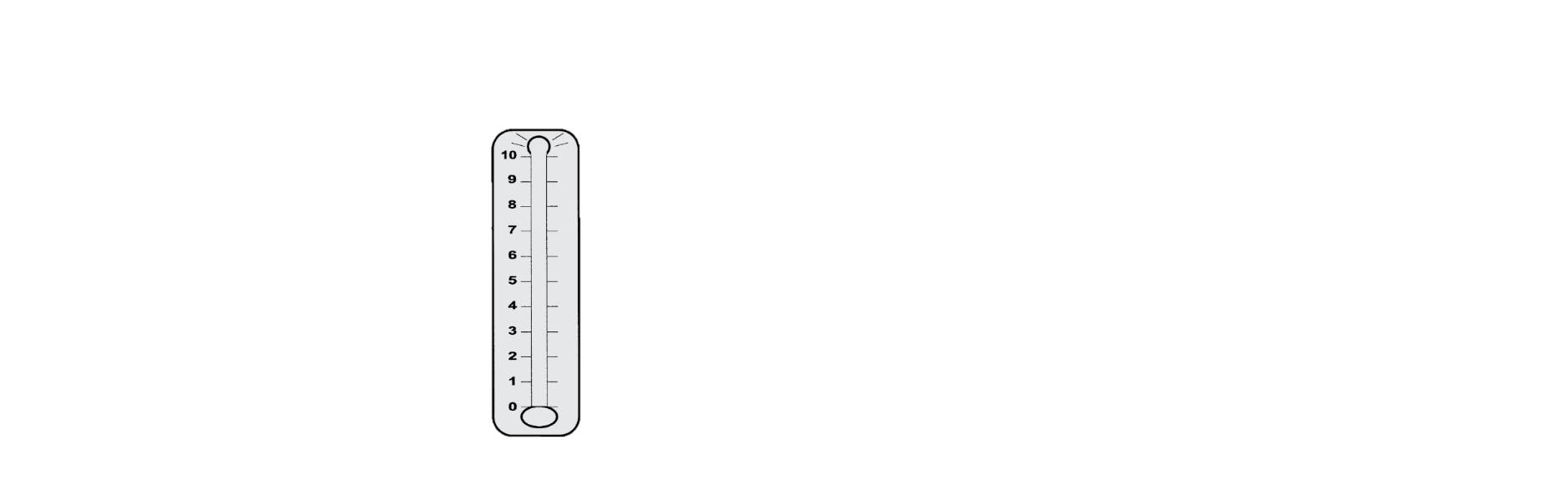Please rate on a scale of 0 to 10 how much distress you experienced due to problems, symptoms, and worries.  **0 is no distress at all, 10 is extreme distress** | … , … | … , … | … , … |

Please note: in case treatment was deemed unnecessary from the Netherlands, you can ignore time point 3 as it does not apply.

You have reached the final part of the questionnaire. The questions are about the preparation for the trip. With this part, we hope to gain insight into the factors that contribute to potential anxiety or psychological distress. This section is therefore purely about identifying factors and not about your actions in the past.

Please note: in case treatment was deemed unnecessary from the Netherlands, you can ignore time point 3 as it does not apply.

|  | | **Time point 1:**  **Moment of departure** | **Time point 2:**  **Directly after the incident** | **Time point 3:**  **Start of treatment** |
| --- | --- | --- | --- | --- |
| 18 | I was aware of the risk of a possible bite or scratch from an animal at my destination | O – Yes  O – No |  |  |
| 19 | I was aware of the fact that rabies exists at my travel destination | O – Yes  O – No  O – Rabies is not endemic at my travel destination |  |  |
|  | If yes:  PrEP was recommended to me | O – Yes  O – No |  |  |
| 20 | I was educated about the disease rabies and what the possible consequences could be when infected | O – Yes  O – No | O – Yes  O – No | O – Yes  O – No |
|  | If yes:  Did you have the idea or feeling ‘that won’t happen to me’? | O – Yes  O – No | O – Yes  O – No | O – Yes  O – No |
| 21 | Which medical help did you receive directly on the spot?  *multiple answers possible |  | O – Nothing  O – Wound cleaning advise  O – Vaccinations  O – Immunoglobulins |  |
| 22 | Which of the following measures did you receive secondarily at your destination?  *multiple answers possible |  |  | O – Nothing  O – Wound cleaning advise  O – Vaccinations  O – Immunoglobulins |
| 23 | I used the internet for additional information about rabies | O – Yes  O – No | O – Yes  O – No | O – Yes  O – No |
| 24 | I was reassured  *multiple answers possible |  | O – Yes, by the local doctor  O – Yes, by Eurocross  O – Yes, by the MHS  O – Yes, by the internet  O – Yes, by the treatment itself (PEP)  O – No | O – Yes, by the local doctor  O – Yes, by Eurocross  O – Yes, by the MHS  O – Yes, by the internet  O – Yes, by the treatment itself (PEP)  O – No |
| 25 | Was the retrieved information (doctor, MHS, internet etc.) about rabies and associated preventive measures reassuring? | O – Yes  O – No | O – Yes  O – No | O – Yes  O – No |
|  | If no:  What was reassuring? | …………………………………  …………………………………  …………………………………  ………………………………… | …………………………………  …………………………………  …………………………………  ………………………………… | …………………………………  …………………………………  …………………………………  ………………………………… |
|  | Explain why. | …………………………………  …………………………………  …………………………………  ………………………………… | …………………………………  …………………………………  …………………………………  ………………………………… | …………………………………  …………………………………  …………………………………  ………………………………… |
|  | Was that a trigger for further action? | O – Yes  O – No | O – Yes  O – No | O – Yes  O – No |
|  | If yes:  What action? | …………………………………  …………………………………  …………………………………  ………………………………… | …………………………………  …………………………………  …………………………………  ………………………………… | …………………………………  …………………………………  …………………………………  ………………………………… |

You have reached the end of the questionnaire. Eurocross Assistance thanks you for your participation.
