# Supplementary material for: The occurrence and extent of anxiety and distress among Dutch travellers after encountering an animal associated injury
Source: Trop Dis Travel Med Vaccines. 2023 Aug 15;9:11. doi: 10.1186/s40794-023-00193-x (PMC10426805; doi:10.1186/s40794-023-00193-x)
Supplement: Supplementary file 2 — Additional file 2. Categorical HADS scores at different time points. [file 40794_2023_193_MOESM2_ESM.docx]

*Additional file 2. Categorical HADS scores at different time points.*

| General Characteristics |  | N (variable) |
| --- | --- | --- |
| HADS score T1  No anxiety (<7), N(%)  Mild anxiety (8-10), N(%)  Moderate anxiety (11-15), N(%)  Severe anxiety (16>), N(%) | 166 (93.8%)  6 (3.4%)  5 (2.8%)  0 (0.0%) | 177 |
| HADS score T2  No anxiety (<7), N(%)  Mild anxiety (8-10), N(%)  Moderate anxiety (11-15), N(%)  Severe anxiety (16>), N(%) | 70 (39.5%)  30 (16.9%)  43 (24.3%)  34 (19.2%) | 177 |
| HADS score T3  No anxiety (<7), N(%)  Mild anxiety (8-10), N(%)  Moderate anxiety (11-15), N(%)  Severe anxiety (16>), N(%) | 95 (65.5%)  15 (10.3%)  21 (14.5%)  14 (9.7%) | 145 |
