# Supplementary material for: The occurrence and extent of anxiety and distress among Dutch travellers after encountering an animal associated injury
Source: Trop Dis Travel Med Vaccines. 2023 Aug 15;9:11. doi: 10.1186/s40794-023-00193-x (PMC10426805; doi:10.1186/s40794-023-00193-x)
Supplement: Supplementary file 3 — Additional file 3. Treatment scheme post-AAI. [file 40794_2023_193_MOESM3_ESM.docx]

*Additional file 3. Treatment scheme post-AAI (2, 5).*

| Type of lesion | PrEP | PEP | Vaccine scheme |
| --- | --- | --- | --- |
| Type I | N/A | N/A | N/A |
| Type II | Yes | 2 RVs | Day 0 and 3 |
|  | No | 4 RVs | Day 0, 3, 7 and between 14 and 28 |
| Type III | Yes | 2 RVs | Day 0 and 3 |
|  | No | 4 RVs | Day 0, 3, 7 and between 14 and 28 |
|  |  | RIG | Day 0 |

*List of abbreviations: PrEP, pre-exposure prophylaxis; PEP, post-exposure prophylaxis; N/A, not applicable; RV, rabies vaccines; RIG, rabies immune globulins.*
